# G protein-coupled receptor kinase 6 is overexpressed in glioma and promotes glioma cell proliferation

**DOI:** 10.18632/oncotarget.17203

**Published:** 2017-04-18

**Authors:** Li-Quan Xu, Shu-Bin Tan, Shan Huang, He-Yuan Ding, Wen-Gang Li, Yi Zhang, Shi-Qi Li, Tao Wang

**Affiliations:** ^1^ Department of Neurosurgery, Shanghai 5th People's Hospital, Shanghai Medical College, Fudan University, Shanghai, 200240, China; ^2^ Department of Endocrinology, Shanghai 5th People's Hospital, Shanghai Medical College, Fudan University, Shanghai, 200240, China; ^3^ Department of Neurosurgery, HuaShan Hospital, Shanghai Medical College, Fudan University, Shanghai, 200040, China

**Keywords:** GRK6, glioma, proliferation, temozolomide

## Abstract

The expression and potential biological functions of G protein-coupled receptor kinase 6 (GRK6) in human glioma are tested in this study. We show that protein and mRNA expression of GRK6 in human glioma tissues was significantly higher than that in the normal brain tissues. Further immunohistochemistry assay analyzing total 118 human glioma tissues showed that GRK6 over-expression was correlated with glioma pathologic grade and patients’ Karnofsky performance status (KPS) score. At the molecular level, in the GRK6-low H4 glioma cells, forced over-expression of GRK6 promoted cell proliferation. Reversely, siRNA-mediated knockdown of GRK6 in the U251MG (GRK6-high) cells led to proliferation inhibition and cell cycle arrest. Intriguingly, GRK6 could also be an important temozolomide resistance factor. Temozolomide-induced cytotoxicity was prominent only in GRK6-low H4 glioma cells. On the other hand, knockdown of GRK6 by targeted siRNA sensitized U251MG cells (GRK6-high) to temozolomide. Thus, GRK6 over-expression in glioma is important for cell proliferation and temozolomide resistance.

## INTRODUCTION

Glioma is the most common malignant tumor in human brain [1]. The most aggressive subtype of glioma is glioblastoma (GBM), which is the grade IV astrocytic tumor [2]. The prognosis of GBM and other advanced glioma has been poor, and the five-year survival is dismal. This is possibly due to tumor cell extremely high proliferation ability and invasiveness trait [3, 4]. Despite the latest developments for glioma treatment, including surgical extirpation, local irradiation, as well as conventional temozolomide (TMZ) chemotherapy [4], the vast majority of patients succumb to the disease within 2 years of diagnosis [5, 6].

G protein coupled receptor kinases (GRKs) are a versatile family of kinases that play a pivotal role in G protein-coupled receptor (GPCR) homologous desensitization [7–9]. GRKs phosphorylate specific serine and threonine residues in the cytoplasmic domains of the activated receptor, thereby promoting receptor interaction, and uncoupling of the receptor from its G protein [7–9]. The GRK family consists of seven members (GRK 1 to 7) [10, 11]. GRK1 and GRK7 are restricted to the visual system; GRK4 is found predominantly in the testis, whereas GRK 2, 3, 5, 6 are expressed in all mammalian cells [9, 10, 12]. Although all GRKs have similar structural organization, they differ in their mechanisms of activation [8, 13–15]. GRK2 and GRK3 are pleckstrin homology (PH) domain-containing proteins, which are recruited to the membrane by Gβγ upon receptor activation [10, 16]. GRK4, GRK5 and GRK6 are membrane-associated proteins, and are directly activated by the receptor and/or ligand complexes [10, 16].

Previous studies have revealed that GRK6 plays an important role in mitotic cell cycle progression [17, 18]. It has been shown that the expression and activity of GRK6 are increased during mitosis [17, 18]. Moreover, dominant negative mutation or knockdown of GRK6 by siRNA induced spindle defects, abnormal chromosome segregation, mitotic arrest and eventually apoptosis [17–19]. These results indicate that GRK6 could be an essential mitotic kinase [17–19]. Several lines of evidence suggested that functional GRK6 is important for tumorigenesis [20, 21]. For example, it was shown that GRK6 mRNA level was upregulated in several tumor cell lines [20, 21]. In the current study, we tested the expression and the potential biological functios of GRK6 in human glioma.

## RESULTS

### GRK6 over-expression in human glioma tissues

**Figure 1:**
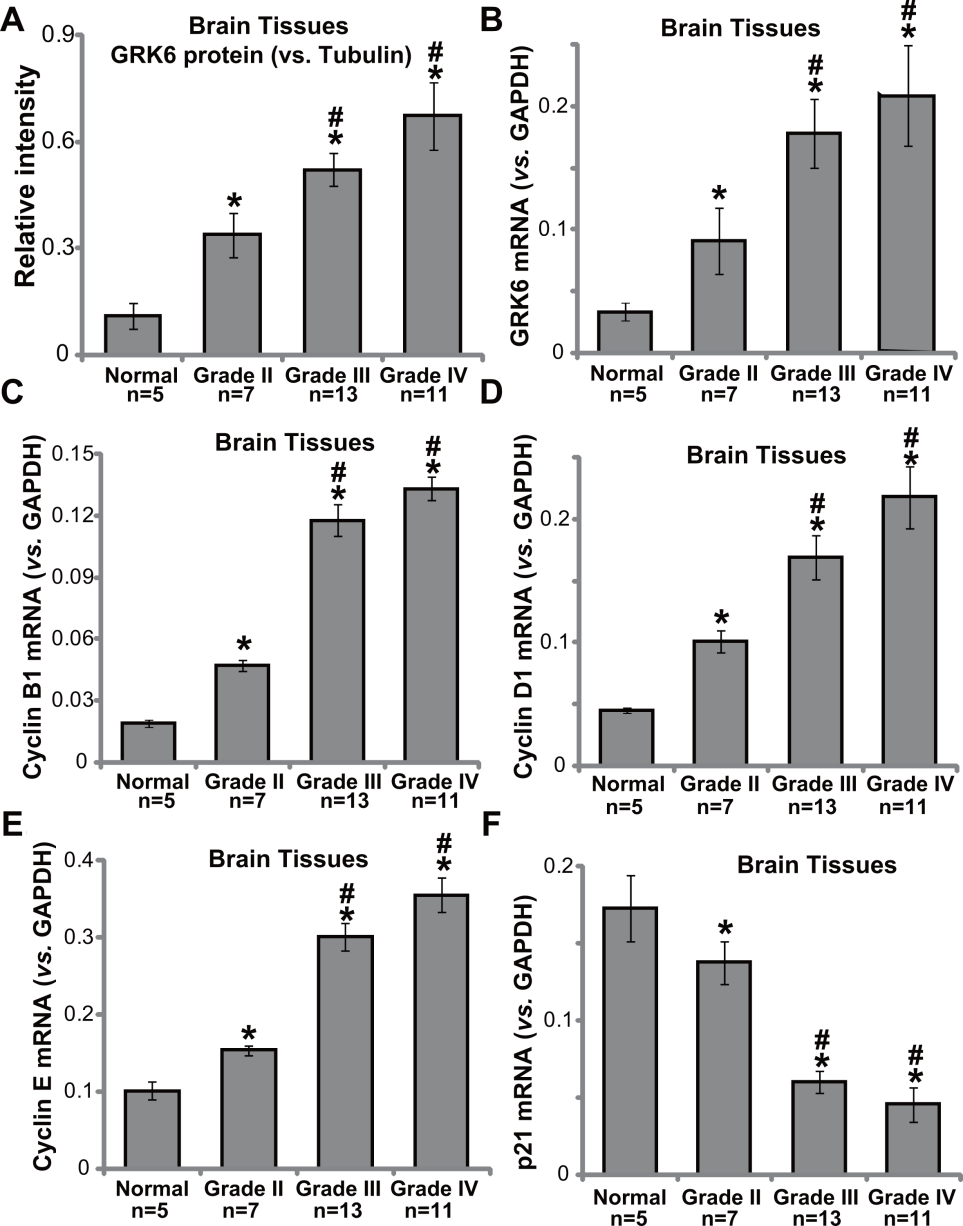
The expression of GRK6 in human glioma tissues Western blotting (**A**) normalized to tubulin) and PT-PCR (**B**) normalized to GAPDH) results showing that the expression of GRK6 was elevated in high-grade glioma tissues (grade III and grade IV), as compared with in low-grade glioma tissues (grade II) and normal brain tissues (“Normal”). Relative mRNA expressions of Cyclin B1 (**C**), Cyclin D1 (**D**), Cyclin E (**E**) and p21 (**F**) were also tested by RT-PCR assay in the tissues. Data were presented as mean ± standard deviation (SD). *P < 0.05 *vs*. “normal brain tissues”. ^#^ *P* < 0.05 *vs*. “grade II glioma tissues”.

First, we tested GRK6 expression in normal brain tissue sample (from epilepsy patients) and various glioma tissue samples (Grade II–IV), all collected freshly at the time of surgery. As shown in Figure 1A and 1B, GRK6 protein and mRNA expression was significantly higher in glioma tissues in comparison with that in the normal brain tissues. Furthermore, quantitative results demonstrated that GRK6 expression (protein and mRNA) level was highest in Grade III/IV glioma tissues, but was less higher in grade II glioma tissues. Its expression was relatively low in the normal brain tissues (Figure 1A and 1B). Intriguingly, GRK6 upregulation in high-grade tumors was correlated with upregulation of multiple proliferation-associated Cyclins, including Cyclin B1 (Figure 1C), Cyclin D1 (Figure 1D) and Cyclin E (Figure 1E). GRK6 expression in above tissues was however negatively correlated with p21 (an anti-proliferative gene) expression (Figure 1F). There results suggest that GRK6 was over-expressed in human glioma tissues, and its upregulation is possible correlated with tumor grade.

### Relationship between glioma patients’ pathologic characteristics and GRK6 expression level

To further test GRK6 expression in human glioma tissues, we examined 118 paraffin-embedded glioma tissues with different histology grades. As presented in Table 1, among the 118 samples, 52 of 118 (44%) cases were identified as low-GRK6 expression, and the other 66 (56%) cases were identified as high-GRK6 expression. Further analysis showed that GRK6 over-expression (“high”) was correlated with the pathologic grade (*P* < 0.05) and Karnofsky performance status (KPS) score (*P* < 0.05) in the glioma patients. We found no significant association between GRK6 expression level and patients’ gender, age, tumor location or extent of resection in the 118 glioma patients (Data not shown).

**Table 1:** GRK6 expression and pathologic variables in 118 glioma specimens

Variables	*n*	GRK6 expression		χ^2^	*P* value
		Low	High		
**KPS**					
**≥ 80**	62	33	29	7.124	< 0.05
**< 80**	52	17	35		
**Unknown**	4	2	2		
**WHO grade**					
**II**	60	17	43	17.553	< 0.05
**III**	38	26	12		
**IV**	20	9	11		

Statistical analyses were performed by the Pearson χ^2^ test.

KPS, Karnofsky Performance Status; WHO, World Health Organization.

*P < 0.05 was considered significant.

### GRK6 expression in human glioma cell lines

**Figure 2:**
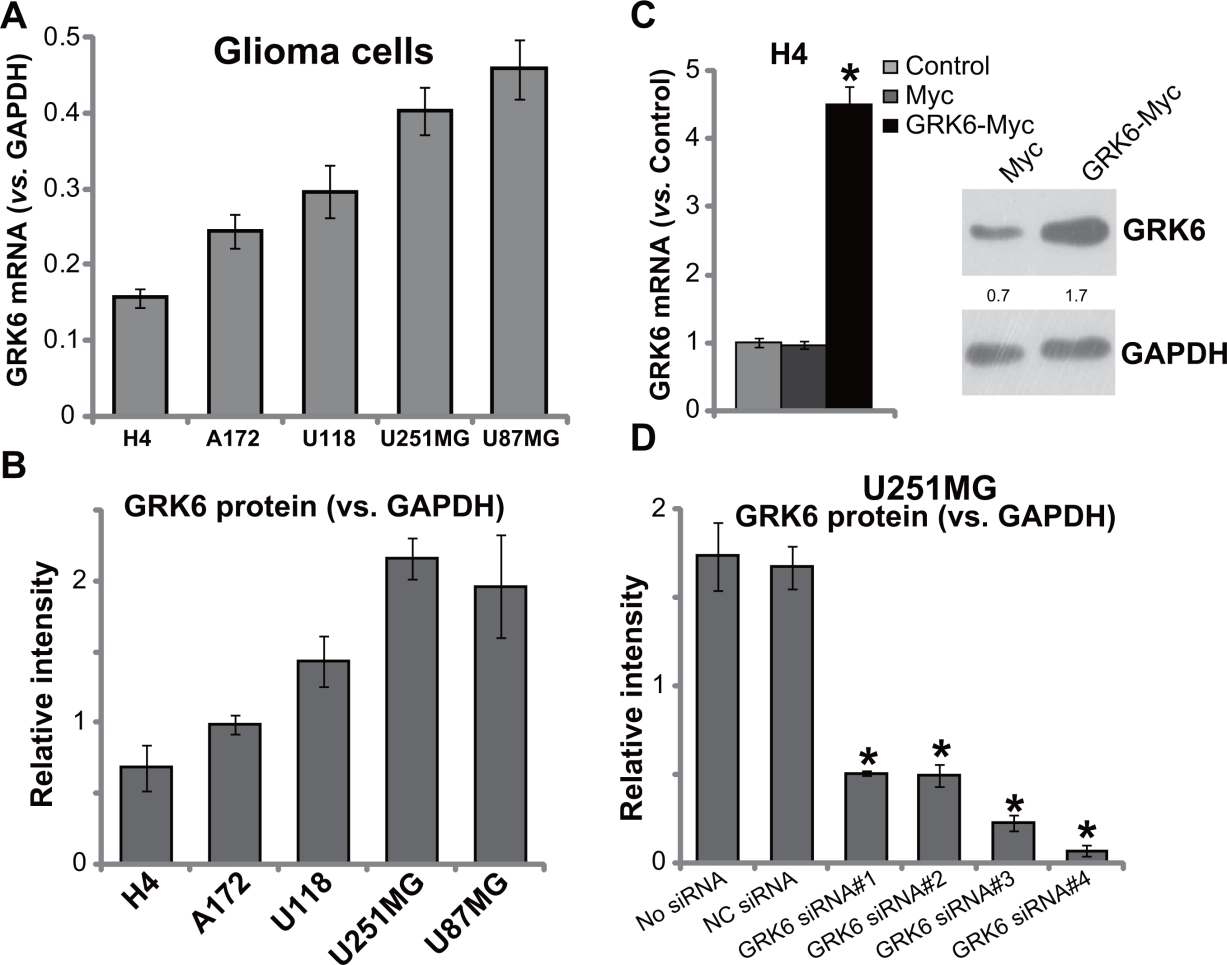
GRK6 expression in human glioma cell lines GRK6 mRNA (**A**) normalized to GAPDH) and protein (**B**) normalized to GAPDH) expression in H4, U118, A172, U251MG and U87MG glioma cells were shown. Expression of GRK6 in the glioma cells with indicated genetic modifications was shown (**C** and **D**). The quantification demonstrated the ratio of GRK6 expression (protein and mRNA) to GAPDH by densitometry. Data were presented as mean ± standard deviation (SD). “Control” stands for un-transfected control cells (C). *P < 0.05 *vs*. “Control” cells (C). “NC siRNA” stands for non-sense control siRNA. Experiments in this figure were repeated four times, and similar results were obtained.

We next tested the expression of GRK6 in several human glioma cell lines, including H4, U118, U251MG, U87MG and A172. RT-PCR assay (Figure 2A) and Western blotting assay (Figure 2B) results showed that U87MG cells and U251MG cells expressed highest level of GRK6. But the H4 glioma cells expressed lowest level of GRK6. To study the potential function of GRK6 in glioma cells, genetic strategies were applied to change GRK6 expression. First, a Myc-GRK6 construct was transfected to the GRK6-low H4 glioma cells. Via puromycin selection, the stable H4 cell line with Myc-GRK6 was established (see Methods). Western blotting assay and RT-PCR assay results in Figure 2C confirmed GRK6 over-expression in the stable H4 cells. On the other hand, a panel of four GRK6 siRNAs (GRK6 siRNA#1-#4) were transfected to the GRK6-high U251MG glioma cells. Quantified Western blotting assay results showed that the applied siRNAs efficiently downregulated GRK6 in U251MG cells (Figure 2D). Of the tested siRNAs, siRNA#3 and siRNA #4 showed highest efficiency in silencing GRK6 (Figure 2D). These two siRNAs were utilized for further experiments.

### GRK6 over-expression facilitates H4 glioma cell proliferation

**Figure 3:**
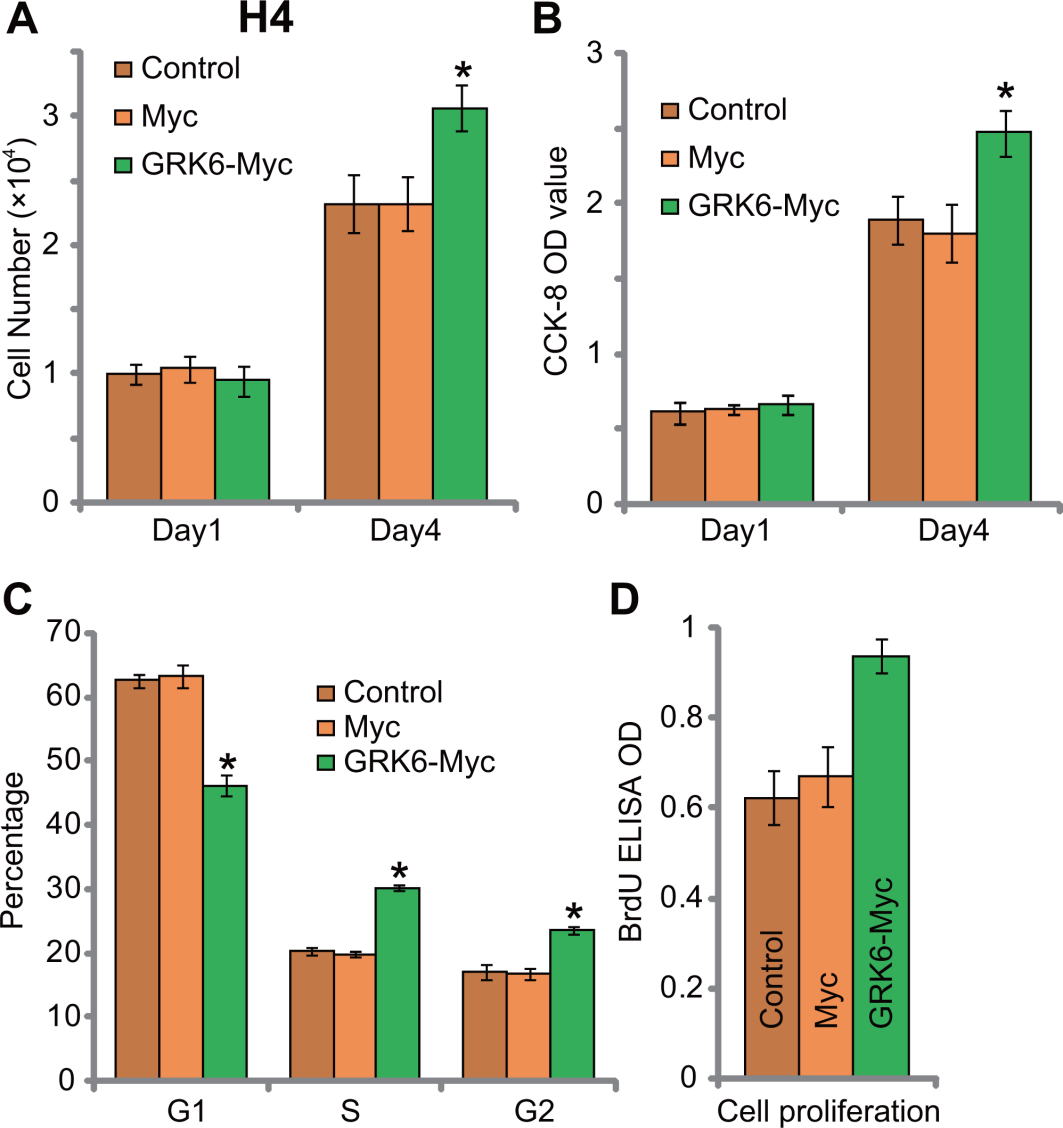
GRK6 over-expression facilitates H4 glioma cell proliferation Un-transfected control (“Ctrl”) H4 cells, Myc- and Myc-GRK6-expressing stable H4 cells were subjected to cell counting assay (**A**), CCK-8 assay (**B**), cell cycle distribution assay (**C**) and BrdU ELISA assay (**D**) of cell proliferation. Data were presented as mean ± standard deviation (SD). *P < 0.05 *vs*. “Control” cells. Experiments in this figure were repeated three times, and similar results were obtained.

Next, cell counting assay and CCK-8 assay were applied to test proliferation. Results in Figure 3A and 3B showed that forced over-expression Myc-GRK6 facilitated H4 glioma cell proliferation. Viable cell number (at Day-4, Figure 3A) and CCK-8 OD (at Day-4, Figure 3B) were both increased after expression of Myc-GRK6. The results from cell cycle analysis displayed that GRK6 over-expression in H4 cells decreased the percentage of cells with G1 phase (Figure 3C), but increased S/G2 phase cells. Further studies showed that GRK6 over-expression in H4 cells increased BrdU ELISA OD (Figure 3D). These results again supported the pro-proliferation activity by GRK6. Thus, over-expression of GRK6 facilitated H4 cell proliferation.

### GRK6 knockdown by siRNA inhibits U251MG glioma cell proliferation

**Figure 4:**
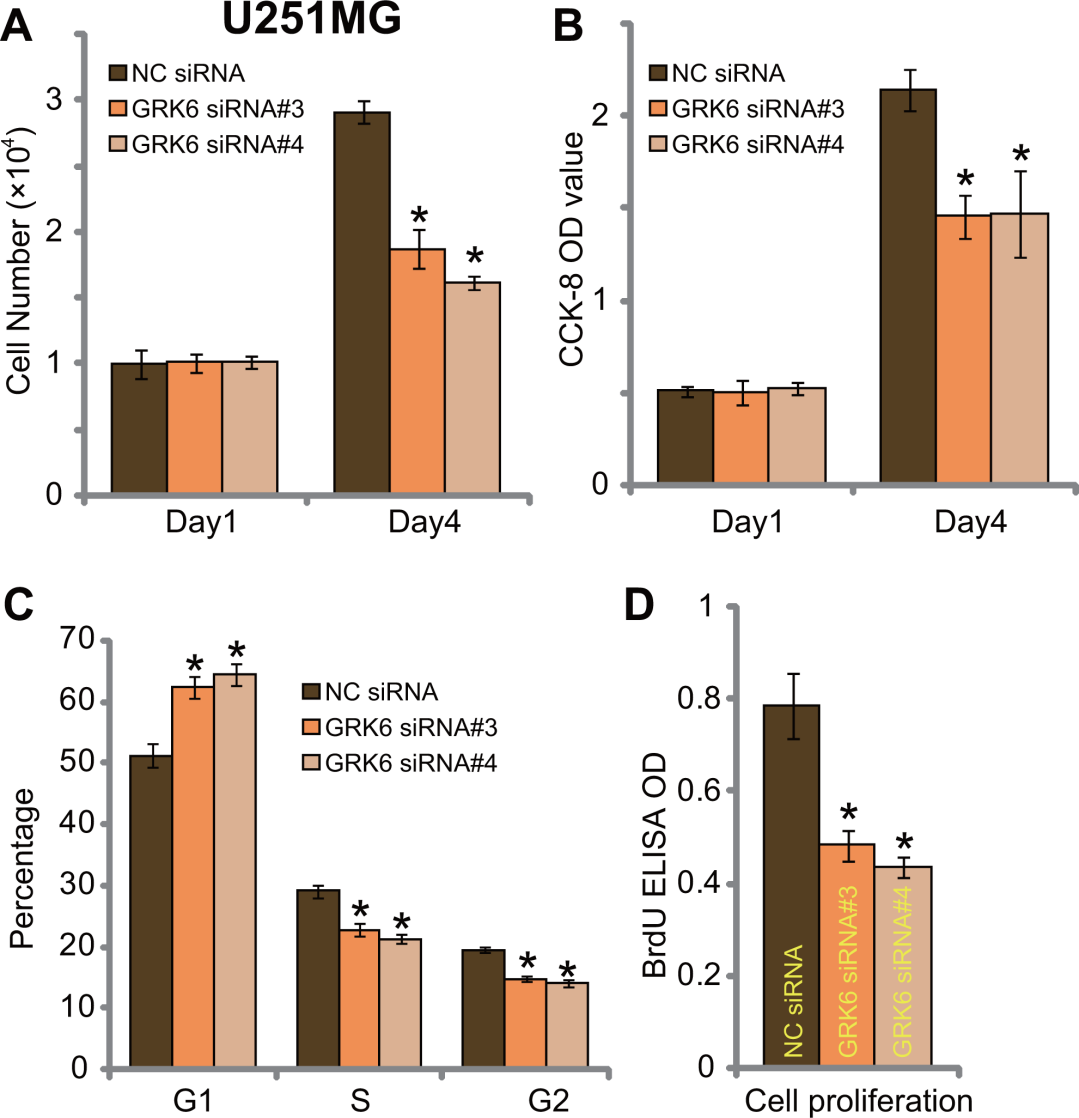
GRK6 knockdown by siRNA inhibits U251MG glioma cell proliferation U251MG cells, transfected with non-sense control siRNA (“NC siRNA”) or GRK6 siRNA (#3 or #4) were subjected the proliferation assays (**A**–**D**). Data were presented as mean ± standard deviation (SD). *P < 0.05 *vs*. “NC siRNA” cells. Experiments in this figure were repeated three times, and similar results were obtained.

On the other hand, siRNA-mediated knockdown of GRK6 inhibited proliferation of U251MG glioma cells (Figure 4A and 4B). Transfection of GRK6 siRNAs (#3 and #4) decreased number of U251MG cells (at Day-4, Figure 4A) and CCK-8 OD (at Day-4, Figure 3B). Meanwhile, GRK6 knockdown in U251MG cells increased the G1 phase cell number, but decreased S/G2 phase cells (Figure 4C), causing G1/S arrest. BrdU ELISA OD was also decreased in the GRK6-silenced U251MG cells (Figure 4D). Together, siRNA-mediated knockdown of GRK6 inhibited U251MG cell proliferation.

### GRK6 knockdown sensitizes human glioma cells to TMZ

**Figure 5:**
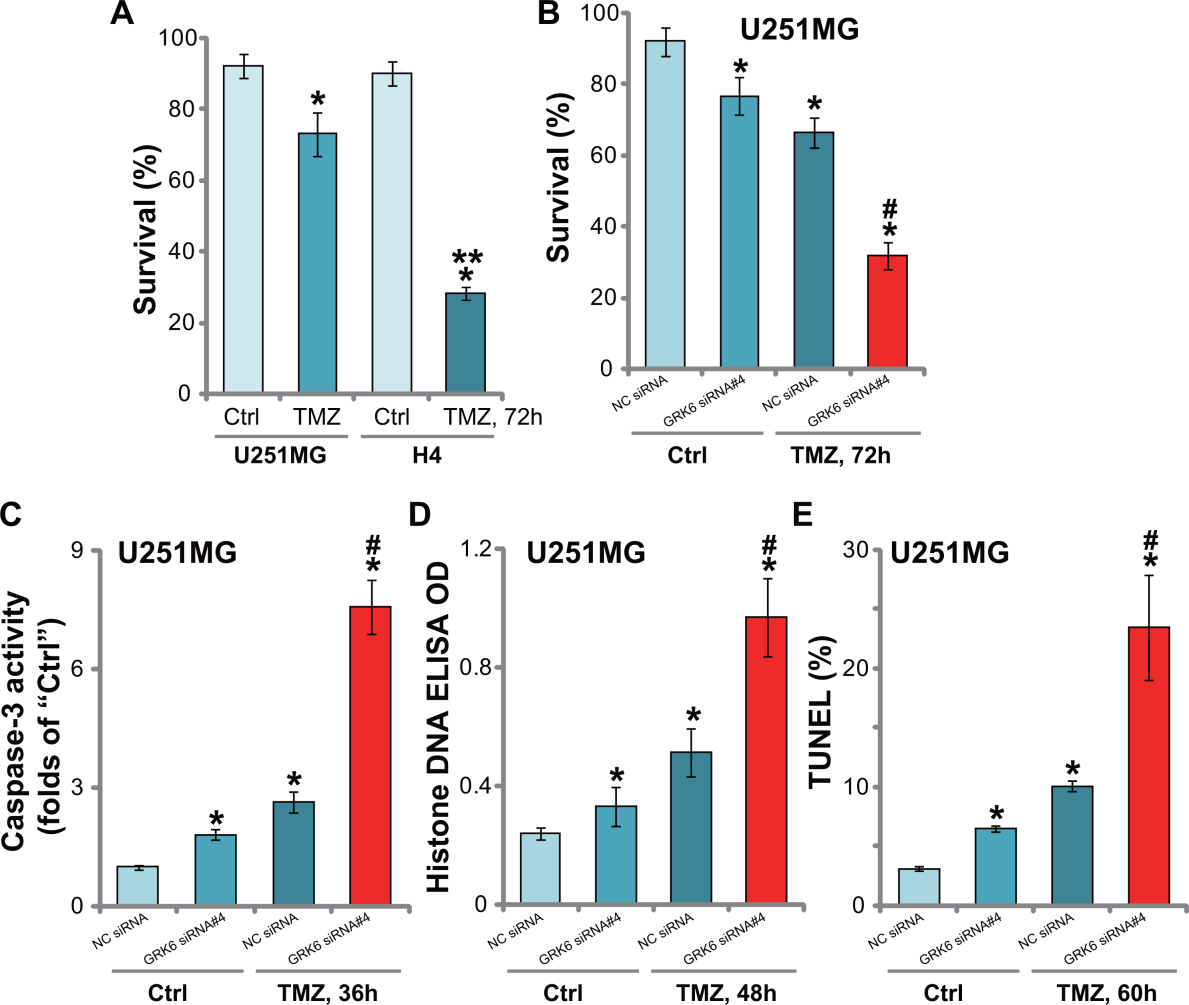
Silencing of GRK6 sensitizes glioma cells to TMZ (**A**) H4 and U251MG cells were treated with TMZ (250 μM) for 72 hours, and cell survival was monitored via the trypan blue staining assay. (**B**) U251MG cells were transfected with the indicated siRNA, and subsequently treated as indicated TMZ (250 μM) for applied time, caspase-3 activity (**C**), Histone DNA apoptosis ELISA OD (**D**) and TUNEL nuclei ratio (**E**) were analyzed. Data were presented as mean ± standard deviation (SD). “Ctrl” stands for untreated control cells. “NC siRNA” stands for non-sense control siRNA. *P < 0.05 *vs*. “Ctrl”. **P < 0.05 *vs*. U251MG cells (A). ^#^*P* < 0.05 *vs*. TMZ treatment of “NC siRNA” cells. Experiments in this figure were repeated three times, and similar results were obtained.

The alkylation agent temozolomide (TMZ) is currently the main chemotherapy agent for glioma treatment [22]. We tested the potential effect of GRK6 on TMZ in glioma cells. Trypan blue survival assay results in Figure 5A showed that GRK6-low H4 cells were sensitive to TMZ (250 μM, 72 hours). On the other hand, the GRK6-high U251MG cells were resistant to the same TMZ treatment (Figure 5A). To further investigate whether GRK6 downregulation could potentiate TMZ's cytotoxicity, U251MG cells were transfected with GRK6 siRNA (#4). Trypan blue staining assay results in Figure 5B showed that GRK6 knockdown by siRNA#4 (See Figure 2) dramatically enhanced TMZ (250 μM)-induced cytotoxicity. GRK6 siRNA alone also induced minor cell death (Figure 5B).

Next, various apoptosis assays, including caspase-3 activity assay (Figure 5C), Histone DNA ELISA assay (Figure 5D) and TUNEL nuclei staining assay (Figure 5E), were applied. Results showed TMZ (250 μM) induced significant apoptosis only in GRK6-silenced U251MG cells (Figure 5C–5E). On the other hand, only minor apoptosis was activated in TMZ-treated control (“NC siRNA”) U251MG cells. Notably, GRK6 siRNA#4 alone also induced moderate U251MG cell apoptosis (Figure 5C–5E). These results implied that GRK6 knockdown remarkably sensitized TMZ-induced cytotoxicity and apoptosis in U251MG cells.

## DISCUSSION

Glioma is the most common and aggressive brain tumor, which has one of the worst prognoses among all malignancies [5]. The median survival of high-grade glioma is approximately 12–14 months [5]. Therefore, identification of genes that are responsible for tumor cell proliferation, migration and invasion is extremely important [1]. In current study, we found that expression of GRK6 in human glioma tissues was significantly higher than that in the normal brain tissues. More importantly, there was a increasing tendency of GRK6 expression in higher grade (Grade III–IV) gliomas. Further, the immunohistochemistry assay results examining GRK6 expression in human glioma tissues indicated that GRK6 expression could be correlated with glioma pathological grade and patients’ KPS score.

One key finding of this study is that GRK6 might be important for glioma cell proliferation. In the GRK6-low H4 glioma cells, forced-overexpression of GRK6 facilitated cell proliferation. Reversely, knockdown of GRK6 in the GRK6-high U251MG cells caused proliferation inhibition and cell cycle arrest. Thus, over-expressed GRK6 in human glioma cells could positively participate in cancer cell proliferation. Further studies will be needed to explore the underlying signaling mechanisms of GRK6 in promoting cell proliferation.

Intriguingly, we show that GRK6 could also be an important TMZ resistance factor in glioma cells. TMZ's cytotoxicity was only prominent in GRK6-low H4 glioma cells, but was relatively weak in GRK6-high U87MG/U251MG cells. Significantly, siRNA-mediated knockdown of GRK6 re-sensitized U87MG cells to TMZ. TMZ-induced cell death and apoptosis were significantly potentiated in U87MG cells with GRK6 silence. Thus, GRK6 knockdown could be another way to increase TMZ's sensitivity in glioma cells.

## MATERIALS AND METHODS

### Primary human glioma specimens and immunohistochemistry analyses

Human glioma tissue specimens (*n* = 118) were collected from enrolled patients at HuaShan Hospital (Shanghai, China) from 2000 to 2006. All tumors were from patients with newly-diagnosed glioma who received no therapy before sample collection. Formalin-fixed, paraffin-embedded sections were prepared for all tissues and reviewed by two independent neuropathologists. Normal brain specimens were acquired from patients undergoing surgery for epilepsy, and were reviewed to verify the absence of tumor. The details of immunohistochemistry methods were described previously [23]. The degree of immuno-staining was reviewed and scored independently by two IHC experts based on the intensity of staining. Staining intensity was graded according to the following criteria: 0 (no staining), 1 (weak staining = light yellow), 2 (moderate staining = yellow brown), and 3 (strong staining = brown). Moderate and strong GRK6 staining were utilized to define tumors with high GRK6 expression, and no and weak staining GRK6 staining were used to indicate low GRK6 expression. Experiments and the protocols requiring clinical samples were approved by the Ethics Committee and Internal Review Board of Fudan University (Shanghai, China). The written-informed consent was obtained from each participant. All investigations were conducted according to the principles expressed in the Declaration of Helsinki.

### Protein extraction and Western blotting assay

Protein samples were prepared by homogenization of fresh human brain tissue specimen in a modified tissue lysis buffer (150 mM NaCl, 50 mM Tris-HCl [pH 7.4], 1 mM phenyl methyl sulphonyl fluoride, 1% Triton X-100, 1% sodium deoxycholic acid, 0.1% sodium dodecyl sulphate [SDS], 5 mg/mL aprotinin and 5 mg/mL leupeptin). The tissue lysates were centrifuged for 10 min at 10,000 rpm to remove tissue debris, and the supernatants were removed and stored. The protein concentration was determined via the BCA Protein Assay Kit (Thermo Scientific Pierce, Rockford, IL). Glioma cells with applied treatment were also incubated with the above lysis buffer. The protein samples (30 μg per lane) were then separated by 10–12% SDS-polyacrylamide gel, and transferred onto a polyvinylidene fluoride (PVDF) membrane. The membrane was then blocked in 5% skim milk in TBST buffer, and was then incubated with primary and corresponding second antibodies. Immuno-reactive bands were visualized by enhanced chemiluminescence reagents (Pierce, Shanghai, China). The band intensity (in total gray) was quantified using the Image J software (NIH).

### Cell culture

The established human glioma cell lines, including H4, U118, U251MG, U87MG, and A172, were purchased from the Cell Library of the Chinese Academy of Sciences (Shanghai, China). Cell lines were cultured in DMEM/RPMI medium (Life Technologies, Shanghai, China) supplemented with 10% fetal bovine serum (FBS, Life Technologies). The medium was changed every 2–3 days. Transient transfection was performed using Lipofectamine 2000 reagent (Life Technologies), according to the manufacturer's recommendations.

### GRK6 siRNA

Small-interfering RNA (siRNA) was designed and synthesized by Nantong Biomics Biotechnologies (Nantong, China). Four siRNAs specifically targeting human GRK6 (Gene ID: 2618 NM_175085.2) were designed and synthesized. The sequence of si-negative control (NC) was also designed by Biomics Biotechnologies. Twenty-four hours prior to transfection, glioma cells were plated with 40–60% confluence. Cells were then transfected by incubation with GRK6 siRNA (NC as a control) at final concentrations of 100 nM with Lipofectamine 2000. Transfection efficiency was verified by Western blotting assay.

### GRK6 over-expression

The human GRK6 cDNA was synthesized by Jikai Biotech (Shanghai, China), which was inserted into the pSuper-puro-Myc vector. Lipofectamine 2000 was applied to transfect the construct to H4 glioma cells. The cells were then subjected to puromycin (5.0 μg/mL) selection for a total of 5–7 passages. Expression of GRK6 in the stable cells was tested by Western blotting assay. Control cells were transfected with empty vector (pSuper-puro-Myc).

### RNA extraction, reverse transcription and PCR

Total RNA from cultured cell and surgically obtained human tissues was extracted using the Trizol reagents according to the manufacturer's instruction. Real-time reverse transcription-polymerase chain reaction (PCR) primers were designed with the assistance of the Primer Express v 2.0 software (Applied BioSystems, Foster, CA). Sequences of the primers are: GRK6, 5′-GCTCCCTTCTTTTAAGGGTTCAA-3′ (forward); GRK6, 5′-ACCAGTAACTGTGACTCCGGT-3′ (reverse); GAPDH, 5′-GACTCATGACCACAGTCCATGC-3′ (forward); GAPDH, 5′-AG-AGGCAGGGATGATGTTCT G-3′ (reverse). Cyclin B1 5′-AGGAAGAGCAAGCAGTC AGAC-3′ (forward), 5′-GCAGCATCTTCTTGGGCAC AC-3′ (reverse); Cyclin D1, 5′-CTGGCCATGAACTAC CTGGA-3′ (forward), 5′-GTCACACTTGATCACTCTG G-3′ (reverse); Cyclin E, 5′-GTTATAAGGGAGACGGGG AG-3′ (forward), 5′-TGCTCTGCTTCTTACCGCTC-3′ (reverse); p21, 5′-AGTGGACAGCGAGCAGCTGA-3′ (forward), 5′-TAGAAATCTGTCATGCTGGTCTG-3′ (reverse). The products were analyzed on a 1.5% agarose gel containing 0.2 μg/mL ethidium bromide, and visualized under an ultraviolet transilluminator. Densitometric analysis of PCR products was performed with ImageJ software, and standardized to the GAPDH product.

### Cell survival assay

Cells were harvested, washed and stained with trypan blue dye (Sigma). Cells with compromised cell membranes took up trypan blue, and were counted as dead cells. Trypan blue negative cells were considered as survival cells.

### CCK-8 cell proliferation assay

Glioma cells were seeded onto 96-well cell culture plates (Corning, Shanghai, China) at a concentration of 2 × 10^4^ cells/well in 100 μL culture medium. Following treatment of cells, proliferation was measured via the commercial Cell Counting Kit (CCK)-8 (Dojindo, Kumamoto, Japan).

### BrdU ELISA assay of cell proliferation

After applied treatment, glioma cells were incubated with BrdU dye (10 μM, Cell Signaling Tech, Shanghai, China) for 12 hours. BrdU incorporation was determined in the ELISA format [24, 25]. ELISA OD was utilized as an indicator or cell proliferation.

### Quantification of apoptosis by ELISA

After applied treatment, cell apoptosis was quantified via the Histone DNA ELISA assay kit (Roche, Shanghai, China). The detailed protocol was described in other studies [26, 27].

### TUNEL assay of apoptosis

After the applied treatment, cell apoptosis was examined by the TUNEL staining assay, the detailed protocol was reported previously [28]. At least 200 cells per treatment in five independent experiments were include to calculate the TUNEL ratio.

### Caspase-3 activity assay of cell apoptosis

The detailed protocol of caspase-3 activity assay was described early [29]. For each preparation, 10 μg of cytosolic protein extracts were added to caspase assay buffer [29] with the caspase-3 substrate [29]. Afterwards, the release of 7-amido-4-(trifluoromethyl)-coumarin (AFC) was quantified through a Fluoroskan system [29]. The optic density (OD) of caspase-3 AFC in the treatment group was always normalized to that of untreated control group.

### Flow cytometry assay of cell cycle

Following the applied treatment, the cells were detached by trypsinization, washed in phosphate-buffered saline (PBS), and fixed in 70% cold ethanol overnight at −20°C. The next day, cells were incubated with RNase solution (100 μg/mL, Sigma) for 30 min at 37°C. Finally, the cells were stained with propidium iodide (100 μg/mL in PBS) at room temperature for 30 min, and analyzed with a Becton-Dickinson flow cytometer BD FACScan (San Jose, CA) [24, 26]. The percentage of cells in the G1, S, and G2-M phases was determined. The experiments were performed in triplicate.

### Statistical analysis

The statistical significance of the means was calculated using Student's *t*-test. Data were presented as mean ± standard deviation (SD). The χ^2^ test was applied to analyze the relationship between GRK6 expression and patients’ clinical features. *P* values of less than 0.05 were required for statistical significance. All computations were carried out using the SPSS 18.0 (SPSS, Inc., Chicago, IL) and Stata 7.0 (Stata Corp LP, College Station, TX).

## CONCLUSIONS

In summary, our results provide new evidence for the involvement of GRK6 in glioma carcinogenesis, and suggest that RNAi-directed GRK6 silencing may be a potent therapeutic strategy for future glioma treatment.
